# Broad-Spectrum Matrix Metalloproteinase Inhibition Curbs Inflammation and Liver Injury but Aggravates Experimental Liver Fibrosis in Mice

**DOI:** 10.1371/journal.pone.0011256

**Published:** 2010-06-25

**Authors:** Vincent E. de Meijer, Deanna Y. Sverdlov, Yury Popov, Hau D. Le, Jonathan A. Meisel, Vânia Nosé, Detlef Schuppan, Mark Puder

**Affiliations:** 1 Department of Surgery and Vascular Biology Program, Children's Hospital Boston, Harvard Medical School, Boston, Massachusetts, United States of America; 2 Department of Surgery, Erasmus Medical Center (MC), University Medical Center Rotterdam, Rotterdam, The Netherlands; 3 Division of Gastroenterology and Hepatology, Beth Israel Deaconess Medical Center, Harvard Medical School, Boston, Massachusetts, United States of America; 4 Department of Anatomic Pathology, Miller School of Medicine, University of Miami, Miami, Florida, United States of America; University Paris Diderot-Paris 7, France

## Abstract

**Background:**

Liver fibrosis is characterized by excessive synthesis of extracellular matrix proteins, which prevails over their enzymatic degradation, primarily by matrix metalloproteinases (MMPs). The effect of pharmacological MMP inhibition on fibrogenesis, however, is largely unexplored. Inflammation is considered a prerequisite and important co-contributor to fibrosis and is, in part, mediated by tumor necrosis factor (TNF)-α-converting enzyme (TACE). We hypothesized that treatment with a broad-spectrum MMP and TACE-inhibitor (Marimastat) would ameliorate injury and inflammation, leading to decreased fibrogenesis during repeated hepatotoxin-induced liver injury.

**Methodology/Principal Findings:**

Liver fibrosis was induced in mice by repeated carbon tetrachloride (CCl4) administration, during which the mice received either Marimastat or vehicle twice daily. A single dose of CCl_4_ was administered to investigate acute liver injury in mice pretreated with Marimastat, mice deficient in *Mmp9*, or mice deficient in both TNF-α receptors. Liver injury was quantified by alanine aminotransferase (ALT) levels and confirmed by histology. Hepatic collagen was determined as hydroxyproline, and expression of fibrogenesis and fibrolysis-related transcripts was determined by quantitative reverse-transcription polymerase chain reaction. Marimastat-treated animals demonstrated significantly attenuated liver injury and inflammation but a 25% increase in collagen deposition. Transcripts related to fibrogenesis were significantly less upregulated compared to vehicle-treated animals, while MMP expression and activity analysis revealed efficient pharmacologic MMP-inhibition and decreased fibrolysis following Marimastat treatment. Marimastat pre-treatment significantly attenuated liver injury following acute CCl_4_-administration, whereas *Mmp9* deficient animals demonstrated no protection. Mice deficient in both TNF-α receptors exhibited an 80% reduction of serum ALT, confirming the hepatoprotective effects of Marimastat via the TNF-signaling pathway.

**Conclusions/Significance:**

Inhibition of MMP and TACE activity with Marimastat during chronic CCl_4_ administration counterbalanced any beneficial anti-inflammatory effect, resulting in a positive balance of collagen deposition. Since effective inhibition of MMPs accelerates fibrosis progression, MMP inhibitors should be used with caution in patients with chronic liver diseases.

## Introduction

Hepatic fibrosis represents the wound healing response to chronic insult and is the final common pathway for most chronic liver diseases, regardless of their mechanism [1]–[3]. Progressive fibrosis ultimately leads to increased mortality and morbidity from portal hypertension, end-stage liver failure and ultimately cirrhosis, and is associated with an increased risk of hepatic malignancies [4]. Currently, the only definitive treatment for advanced fibrosis and cirrhosis is liver transplantation; however, the demand for organ grafts outweighs their availability [5], stressing the need for effective antifibrotic approaches [6], [7].

Hepatocellular injury usually leads to inflammation and activation of the innate immune system, leading to release of growth factors, cytokines and small molecular mediators that can stimulate extracellular matrix (ECM) synthesis by activation of quiescent hepatic stellate cells and fibroblasts/myofibroblasts (collectively named HSCs) [1], [2]. Upon fibrogenic activation, HSCs as well as inflammatory cells release and respond to the cytokine transforming growth factor (TGF)-β [8]. TGF-β strongly upregulates production and deposition of the major ECM constituents, while it downregulates fibrolytic matrix metalloproteinases (MMPs) [8], [9]. In the presence of chronic hepatic injury, an imbalance between fibrogenesis and fibrolysis may lead to excess ECM deposition and scar formation.

Cell surface-bound and soluble MMPs along with their endogenous tissue inhibitors (TIMPs) constitute an important system for regulating ECM turnover; however, MMPs also regulate inflammatory processes [10]. Chronic inflammation is an important driver in fibrogenesis, serving both as a trigger and perpetuator of fibrosis progression [11]. A critical mediator of the inflammatory response is tumor necrosis factor (TNF)-α, which exists in a biologically active, soluble form and as an inactive, membrane-anchored precursor [12]. Cleavage of the TNF-α proform into its soluble form is mediated by TNF-α-converting enzyme (TACE, also known as ADAM17 and CD156b), which belongs to the disintegrin and metalloproteinase (ADAM) family of zinc-metalloproteinases [13], [14]. Mice deficient in TIMP3, the endogenous physiological inhibitor of TACE [15], demonstrate elevated levels of TNF-α and develop severe inflammation of the liver, presumably due to depressed TACE activity [16]. In contrast, pharmacologic TACE-inhibition abrogates the inflammatory response and has been demonstrated to have therapeutic potential in a variety of pathological conditions [17], [18]. Many TACE-inhibitors, however, are relatively non-specific and also inhibit various MMPs.

MMPs are widely believed to be important players in fibrosis due to their collagen-cleaving activity [19]–[21]. Identification of novel MMP substrates, however, revealed their involvement in highly complex processes such as the regulation of cell behavior, cell-cell communication, and tumor progression [22], [23]. Hence, these insights indicate that MMPs have a much more complex function in fibrosis than merely ECM degradation. Effects of MMP-inhibition on fibrogenesis, however, remain to be established. We hypothesized that treatment with a broad-spectrum MMP and TACE-inhibitor would ameliorate both injury and inflammation, resulting in decreased fibrosis formation in a murine model of repeated carbon tetrachloride (CCl_4_) administration.

## Results

### Chronic broad-spectrum MMP-inhibition dramatically reduces histological liver injury in mice subjected to chronic CCL_4_-intoxication

**Figure 1:**
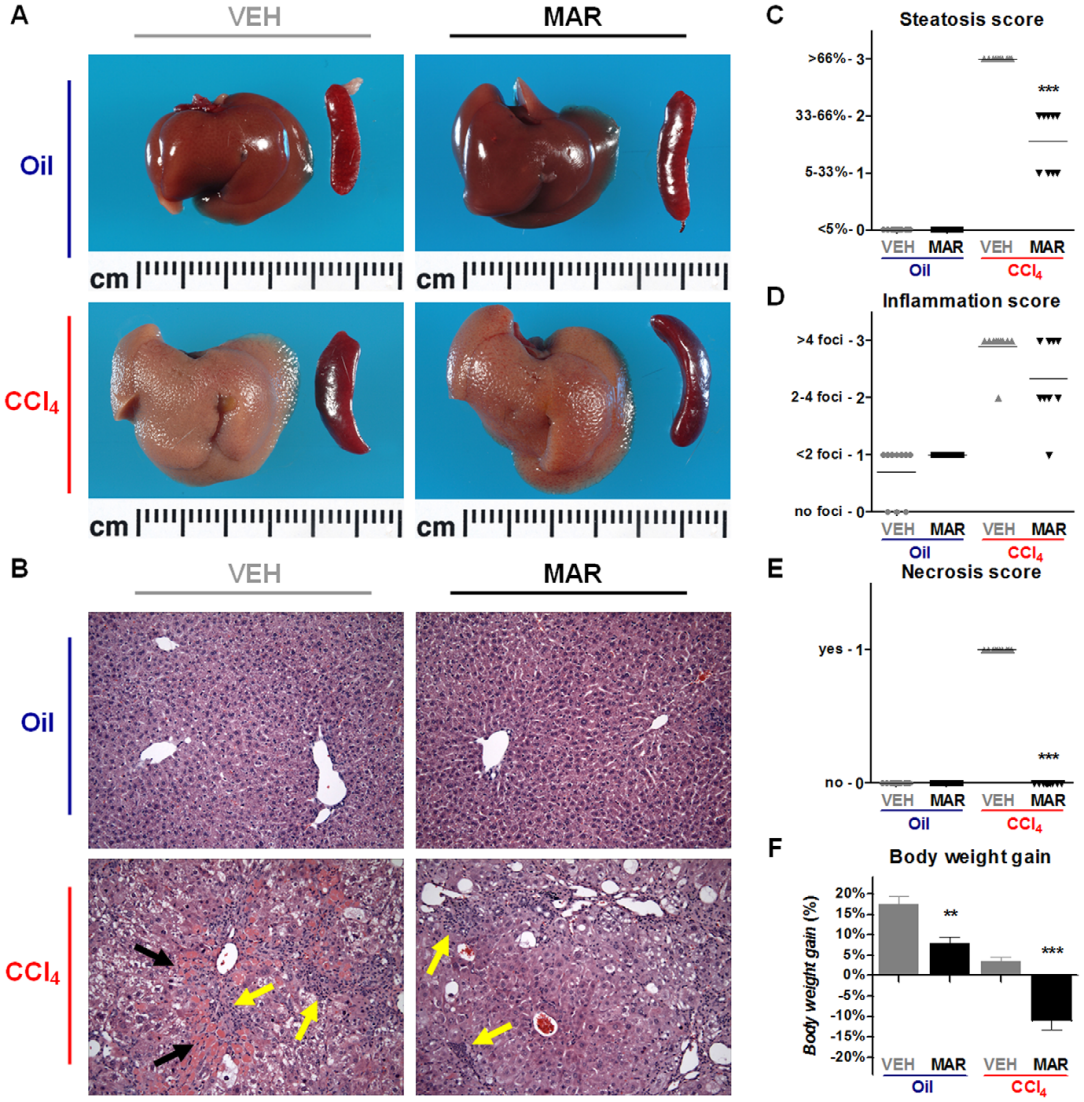
Marimastat treatment reduced liver injury, necrosis, and inflammation following repeated carbon tetrachloride (CCl4) administration. Chronic CCl4 administration resulted in liver enlargement and fibrosis (A). Hematoxylin and eosin staining of liver sections revealed decreased steatosis and inflammation (yellow arrows), and no evidence of necrosis (black arrows) in the Marimastat treated mice (B). On liver sections scored by a blinded pathologist and compared to vehicle treated controls, Marimastat treated animals showed a significantly lower steatosis score (C), less inflammatory foci per 200× field (D) and essentially no evidence of necrosis was observed (E); despite body weight loss (F). Oil, non-fibrotic control group; CCl4, fibrotic mice; VEH, vehicle treated control group; MAR, Marimastat treated experimental group; ***, P<0.001 vs. vehicle alone. Data are expressed as means ± standard error. Original magnification: 200×.

Chronic CCl_4_-administration resulted in liver enlargement and fibrosis (**Figure 1A**). Liver sections of vehicle treated controls exhibited areas of necrosis, steatosis, and inflammatory lymphocytic infiltrates –hallmarks of severe chronic hepatic injury (**Figure 1B**). Liver sections from Marimastat treated animals, however, showed a significant reduction in steatosis (**Figure 1C**), inflammation (**Figure 1D**) and necrosis (**Figure 1E**), suggesting attenuation of hepatic injury and inflammation, despite a loss of body weight (**Figure 1F**).

### Marimastat treatment markedly blunts the increase of serum ALT and levels of TNF-α receptor II in CCl_4_-induced chronic hepatic injury

**Figure 2:**
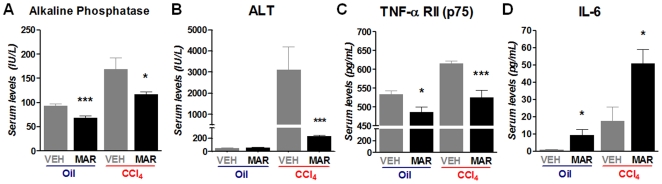
Marimastat treatment ameliorated hepatic injury and the inflammatory response following repeated carbon tetrachloride (CCl4) administration. Marimastat treatment significantly reduced serum alkaline phosphatase levels (A), and resulted in a 14-fold decrease of serum ALT (B), indicating decreased hepatic injury. Serum TNF-α receptor II (p75) levels as measured by ELISA decreased following Marimastat treatment, suggesting successful inhibition of TNF-α converting enzyme (TACE) and an ameliorated inflammatory response. IL-6 serum levels as measured by ELISA increased following Marimastat treatment, suggesting hepatoprotection and stimulated liver regeneration (D). Oil, non-fibrotic control group; CCl4, fibrotic mice; VEH, vehicle treated control group; MAR, Marimastat treated experimental group; ALT, alanine aminotransferase; TNF, tumor necrosis factor; IL, interleukin; *, P<0.05; ***, P<0.001 vs. vehicle alone. Data are expressed as means ± standard error.

Marimastat treatment resulted in a 1.4-fold reduction of alkaline phosphatase levels (*P*≤0.05, **Figure 2A**) and a 14-fold decrease in serum ALT levels (*P*≤0.05, **Figure 2B**), indicating markedly decreased hepatic injury following repeated CCl_4_-administration. Serum levels of soluble TNF-α receptor II (p75) were 1.2-fold decreased in Marimastat treated animals (*P*≤0.05), likely reflecting pharmacologic inhibition of TACE and an ameliorated inflammatory response (**Figure 2C**) [22], [24]. Serum IL-6 levels were increased 2.9-fold (*P*≤0.05), suggesting hepatoprotection and stimulated liver regeneration (**Figure 2D**) [25].

### Marimastat treatment leads to downregulation of major pro-fibrogenic genes

**Figure 3:**
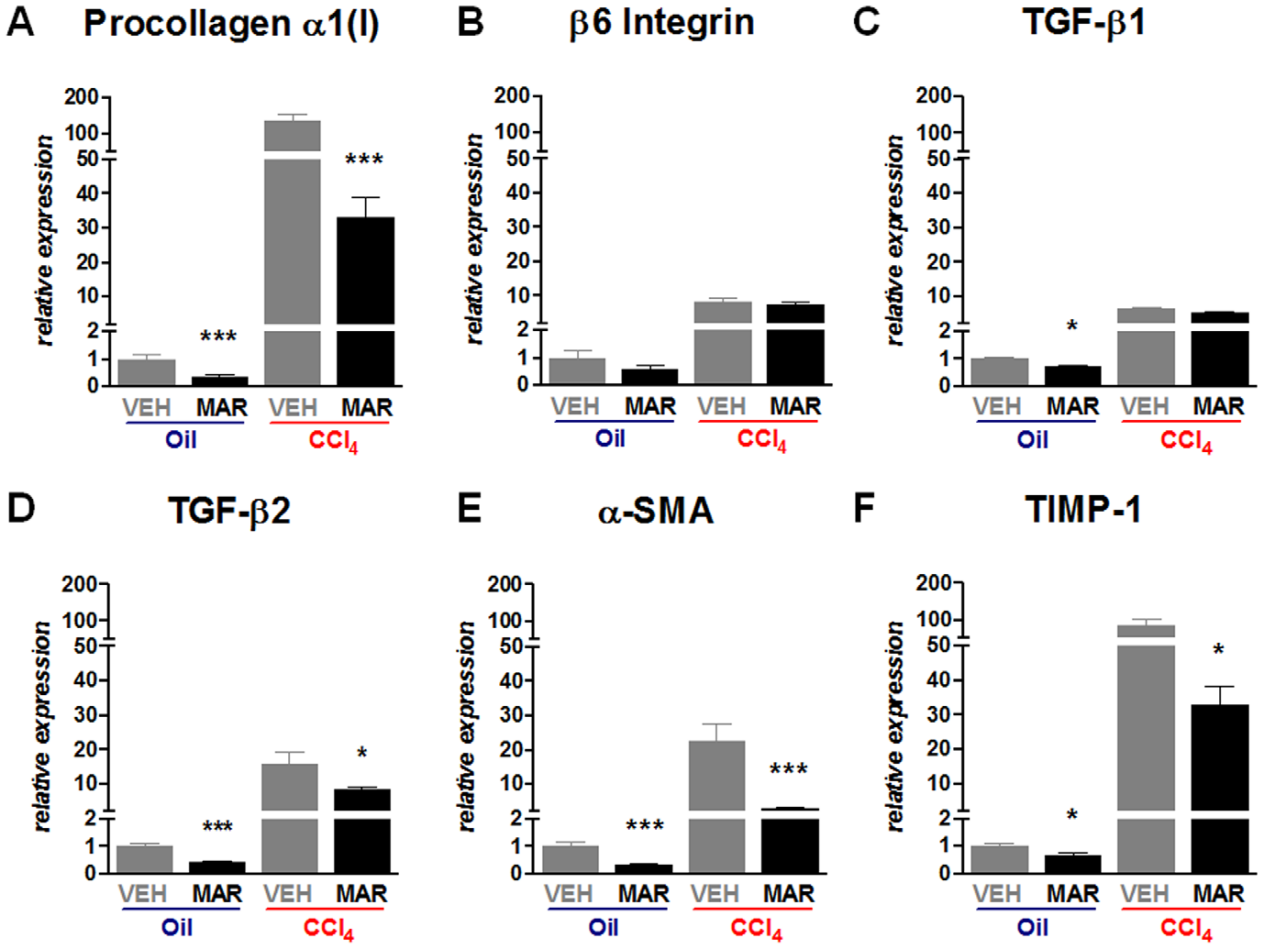
Marimastat altered the hepatic fibrosis-related gene expression profile by downregulation of multiple pro-fibrogenic transcripts following repeated carbon tetrachloride (CCl4) administration. Hepatic procollagen α1(I) (A), β6 integrin (B), TGF-β1 (C), TGF-β2 (D), α-SMA (E) and TIMP-1 (F) expression as quantified by real-time RT-PCR in total liver RNA. Oil, non-fibrotic control group; CCl4, fibrotic mice; VEH, vehicle treated control group; MAR, Marimastat treated experimental group; TGF, transforming growth factor; SMA, smooth muscle actin; TIMP, tissue inhibitor of metalloproteinases; *, P<0.05; ***, P<0.001 vs. vehicle alone. Data are expressed as means ± standard error and in arbitrary units relative to non-fibrotic vehicle treated control.

Hepatic expression of procollagen α1(I), β6 Integrin, TGF-β1, TGF-β2, alpha-smooth muscle actin (α-SMA) and TIMP-1 mRNA were strongly upregulated following repeated CCl_4_-administration (**Figure 3**). Concomitant treatment with Marimastat, however, significantly decreased hepatic transcript levels of procollagen α1(I) (**Figure 3A**), TGF-β2 (**Figure 3D**), α-SMA (**Figure 3E**) and TIMP-1 (**Figure 3F**) compared to vehicle treated controls, whereas β6 Integrin (**Figure 3B**) and TGF-β1 (**Figure 3C**) mRNA levels remained unchanged.

### Marimastat does not diminish net collagen deposition and fibrosis formation

**Figure 4:**
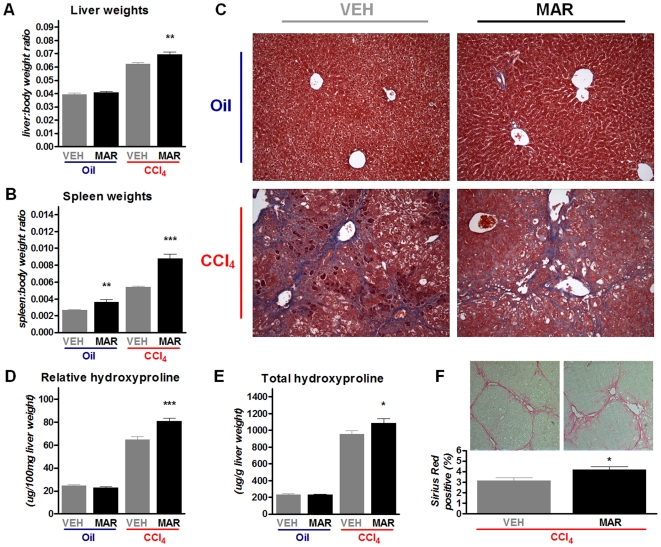
Marimastat treatment increased hepatic fibrosis following repeated carbon tetrachloride (CCl4) administration. In mice treated with Marimastat, the liver to body weight ratio (A), as well as the spleen to body weight ratio (B) were increased. Masson trichrome staining of liver sections for collagen (blue; C) revealed bridging portal fibrosis. Livers from Marimastat treated animals showed occasional focal cirrhosis; however, advanced fibrosis was predominant (C). Livers from Marimastat treated mice exhibited increased collagen deposition as determined biochemically as relative hydroxyproline content and total hydroxyproline content in liver samples from two different lobes (D, E). Relative fibrotic area was increased in livers from mice treated with Marimastat, as quantified using morphometric analysis of Sirius Red stained liver sections (F). Oil, non-fibrotic control group; CCl4, fibrotic mice; VEH, vehicle treated control group; MAR, Marimastat treated experimental group; *, P<0.05; **, P<0.01; ***, P<0.001 vs. vehicle alone. Data are expressed as means ± standard error.

Treatment of mice with Marimastat during chronic CCl_4_-administration significantly increased the liver and the spleen (a putative marker of portal hypertension) to body weight ratios, compared to controls (**Figure 4A, B**). Liver sections of the vehicle treated controls exhibited centrilobular fibrosis with areas of necrosis, whereas liver sections from Marimastat treated animals showed enhanced centrilobular collagen deposition indicating increased fibrosis formation (**Figure 4C**). To directly quantify the degree of fibrosis, we measured both relative (per g of liver) and total (per whole liver) collagen content biochemically via hepatic hydroxyproline determination. Marimastat treatment resulted in a significant increase in relative and total collagen (hydroxyproline) content (25% and 14%, respectively) compared to the controls (**Figures 4D, E**). This was corroborated using morphometric analysis of Sirius Red stained liver sections (**Figure 4F**), demonstrating that the relative fibrotic area was significantly increased in livers from mice treated with Marimastat, compared to controls (4.2% versus 3.1%, respectively; *P*<0.05). These results suggest dissociation between hepatic injury and inflammation on the one hand, and the degree of fibrosis on the other hand, upon pharmacological MMP inhibition.

### Marimastat decreases hepatic stellate cell activation, but increases recruitment of inflammatory cells

**Figure 5:**
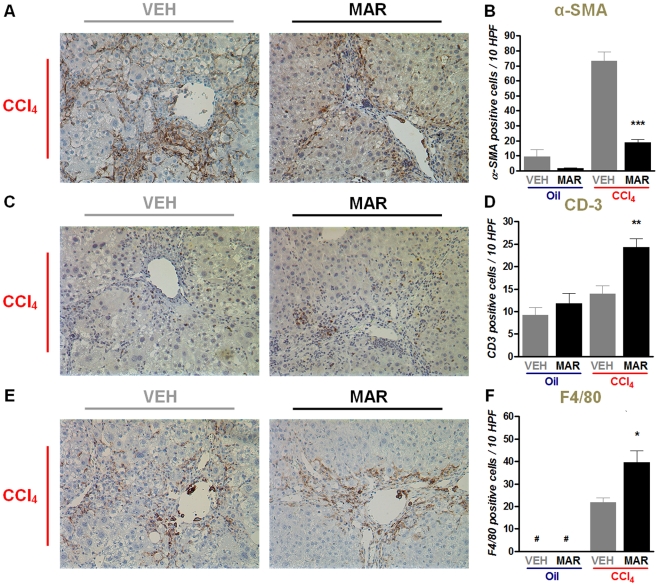
Marimastat decreases hepatic stellate cell (HSC) activation, but increases recruitment of inflammatory cells. Chronic carbon tetrachloride (CCl4) administration in animals treated with Marimastat resulted in a decreased activation of HSCs, as identified by alpha-smooth muscle actin (α-SMA) staining (A). Quantification revealed that following chronic CCl4 administration, Marimastat treated animals had a 74% decrease of activated HSCs, compared to controls (B). Liver sections from animals that were chronically challenged with CCl4 showed that resident T cells (CD3, C,D) and macrophages (F4/80, E,F) counts increased up to 2-fold upon Marimastat treatment. Oil, non-fibrotic control group; CCl4, fibrotic mice; VEH, vehicle treated control group; MAR, Marimastat treated experimental group; α-SMA, alpha-smooth muscle actin; *, P<0.05; **, P<0.01; ***, P<0.001 vs. vehicle alone. Data are expressed as means ± standard error. Original magnification: 200×.

Immunohistochemical staining for α-SMA (**Figure 5A**), as a marker for activated HSCs, was performed to confirm the observed decrease in α-SMA mRNA (**Figure 3E**). α-SMA positive cells were significantly increased following chronic CCl_4_-administration; however, concomitant treatment with Marimastat significantly decreased this number indicating decreased activation of a subset of activated HSCs (**Figure 5A,B**). This again suggests that Marimastat treatment led to a decrease in fibrogenic activity, which is in dissociation with the observed, net result of increased fibrosis (**Figure 4**). To further explore this finding, immunohistochemical staining for T-cells and macrophages (i.e., Kupffer cells) was performed. Chronic administration of CCl4 induces an inflammatory response, elicited by accumulation of T-cells and macrophages that remove injured hepatocytes and stimulate fibroblast function [26]. Immunohistochemical staining for CD3 (**Figure 5C**), as a marker for T-cells, revealed a 1.8-fold increase in cell counts in Marimastat-treated animals that were subjected to chronic CCl_4_-administration (**Figure 5D**). In addition, cell counts from F4/80 positive cells, as a marker for macrophages, revealed a 1.8-fold increase following treatment of mice with Marimastat during chronic CCl_4_-administration (**Figure 5E,F**).

### Marimastat treatment downregulates MMP gene expression and MMP-activities

**Figure 6:**
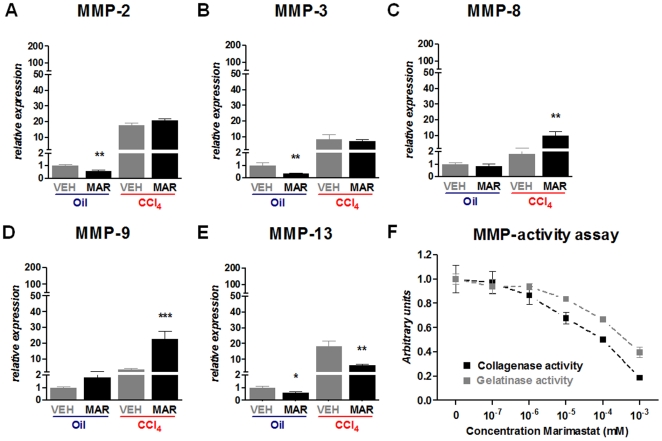
Marimastat treatment downregulates matrix metalloproteinase (MMP) gene expression and MMP-activities. Following repeated carbon tetrachloride (CCl4) administration, Marimastat did not affect hepatic transcript levels of MMP-2 (A), or MMP-3 (B) as quantified by real-time RT-PCR. Hepatic MMP-8 (C) and MMP-9 (D) transcripts were significantly upregulated in livers from Marimastat treated animals, whereas hepatic MMP-13 mRNA was significantly higher in the vehicle treated animals (E). Marimastat inhibits interstitial collagenolytic and gelatinolytic activity in a dose-dependent matter (F). Relative gelatinase and interstitial collagenase activities after 4 hours in liver homogenates supplemented with increasing concentrations of Marimastat, as determined by degradation of DQ-gelatin and collagen (black and grey squares, respectively). Oil, non-fibrotic control group; CCl4, fibrotic mice; VEH, vehicle treated control group; MAR, Marimastat treated experimental group; TNF, tumor necrosis factor; MMP, matrix metalloproteinase; *, P<0.05; **, P<0.01; ***, P<0.001 vs. vehicle alone. Data are expressed as means ± standard error and in arbitrary units.

To better understand the increased ECM deposition in animals treated with Marimastat, we analyzed hepatic MMP expression levels. In fibrotic animals, hepatic expression of MMP-2, MMP-3, MMP-8, MMP-9 and MMP-13 mRNA were all significantly upregulated, compared to non-fibrotic controls (**Figure 6**). Marimastat treatment during chronic CCl_4_ administration, however, did not affect hepatic transcript levels of MMP-2 (**Figure 6A**), or MMP-3 (**Figure 6B**), compared to vehicle treated animals. Hepatic transcript levels of MMP-8 (**Figure 6C**) and MMP-9 (**Figure 6D**) were 5.6-fold and 6.6-fold increased, respectively, in livers from Marimastat treated animals, whereas hepatic MMP-13 transcripts were 3.0-fold higher in the vehicle treated animals (**Figure 6E**). Increased MMP expression levels may have resulted from a positive feedback mechanism resulting from efficient pharmacologic inhibition of MMP-activity, although this would not explain downregulation of MMP-13 [22]. We therefore performed an assay to investigate the ability of Marimastat to inhibit gelatinase (MMP-2, MMP-9) and interstitial collagenase (MMP-1) activities. Relative collagenase activity as well as relative interstitial gelatinase activity in liver homogenates supplemented with increasing concentrations of Marimastat showed a dose-dependent inhibition of both these MMP-activities (**Figure 6F**). These findings indicate efficient inhibition of putatively fibrolytic MMPs by Marimastat, potentially leading to increased fibrosis in the presence of continuous hepatic injury.

### Broad spectrum MMP-inhibition with Marimastat using a model of acute administration of a single dose of CCl_4_ predicts the evolution of the chronic CCl_4_ model

**Figure 7:**
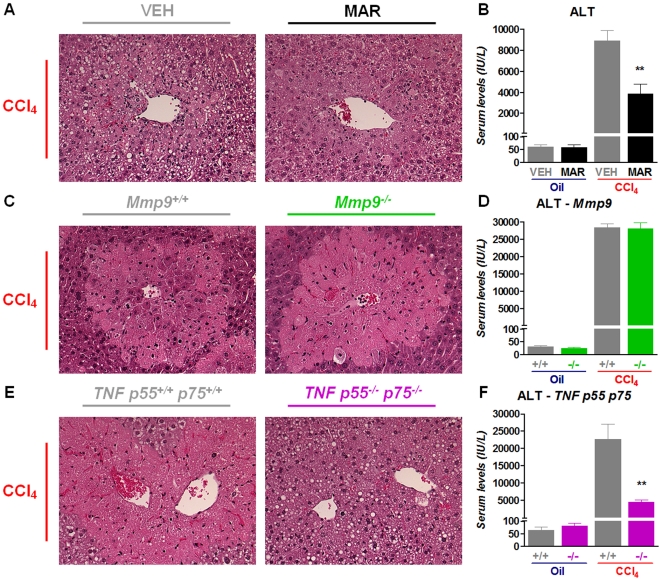
Marimastat reduces necroinflammatory injury following a single dose of carbon tetrachloride (CCl4) via a TNF-dependent pathway. Liver sections from Marimastat-treated animals that had been administered a single dose of CCl4 revealed decreased necroinflammatory injury after 24 h (A). Marimastat treatment resulted in a 57% decrease of serum ALT levels (B). Liver sections from Mmp9−/− and wild type (Mmp9+/+) animals that were challenged with a single dose of CCl4 showed comparable, extensive necroinflammatory changes around the central veins after 24 h (C). Serum ALT levels of Mmp9−/− animals were similar to those of wild type controls (D). 24 h after a single dose of CCl4, liver sections from wild type (TNF p55+/+ p75+/+) animals revealed extensive necrosis and inflammation around the central veins, whereas TNF p55−/− p75−/− animals showed markedly reduced hepatic injury (E). Serum ALT levels in TNF p55−/− p75−/− animals were 5-fold lower compared to wild type controls, corroborating the findings on histology and indicating decreased hepatic injury in animals lacking both TNF-receptors (F). Oil, non-fibrotic control group; CCl4, fibrotic mice; VEH, vehicle treated control group; MAR, Marimastat treated experimental group; +/+, wild type controls; −/−, knock out animals; ALT, alanine aminotransferase; TNF, tumor necrosis factor; **, P<0.01 vs. vehicle alone. Data are expressed as means ± standard error. Original magnification: 300×.

To further explore the anti-inflammatory and hepatoprotective potential of pharmacologic broad-spectrum MMP and TACE inhibition, C57Bl/6J mice were pretreated for 7 days with Marimastat and subsequently challenged with a single dose of CCl_4_. Analysis of liver sections at 12 h, 24 h, 48 h and 96 h time points revealed that acute CCl_4_ intoxication resulted in severe necroinflammatory injury around the central vein areas with a peak at 24 h, regeneration and repair at 48 h characterized by influx of macrophages and inflammatory lymphocytic infiltrates, and impressive resolution of necrotic injury after 96 h (data not shown). After 24 h, Marimastat pre-treatment attenuated necroinflammatory hepatic injury as determined by histology (**Figure 7A**), and resulted in a 57% reduction in serum ALT levels (*P*≤0.05, **Figure 7B**). Since the peak of hepatic injury occurred after 24 h, this time point was chosen as surrogate endpoint in subsequent experiments.

### Marimastat reduces necroinflammatory injury following a single dose of CCl_4_ via a TNF-dependent pathway

Next, we studied if the anti-inflammatory and hepatoprotective effects of Marimastat could be attributed to either an MMP- or a TACE- dependent mechanism by using gene deleted animals. MMP-9 is the major and most studied and abundant MMP in inflammation [10], [27], playing a critical role in fulminant TNF-mediated hepatitis [28], mediating hepatic ischemia/reperfusion injury [29] and being involved in liver regeneration [30]. Given that Marimastat efficiently inhibits MMP-9 activity (**Figure 6**), *Mmp9* homozygous null mice (*Mmp9^−/−^*) and their WT littermates were subjected to a single dose of CCl_4_, or vehicle (mineral oil) to investigate the involvement of a single MMP, rather than multiple MMPs by pharmacologic broad-spectrum MMP-inhibition on CCl_4_-induced hepatotoxicity. After 24 h liver sections from WT animals and *Mmp9^−/−^* animals exhibited extensive centrilobular necroinflammatory changes (**Figure 7C**). Serum ALT levels were 28,410 IU/L in WT animals compared to 28,100 IU/L in *Mmp9^−/−^* animals (*P* = 0.89; **Figure 7D**). Taken together, these findings do not indicate that MMP-9 plays a significant role in the acute phase of CCl_4_-induced hepatic injury.

The ability of Marimastat to inhibit TACE activity prompted us to study the role of TNF-α signaling in CCl_4_-induced acute hepatotoxicity. Since TACE is one of the major activators of TNF-α, TACE-inhibition has been implicated as a promising anti-inflammatory approach [17], [24]. Because homozygous TACE deletion is embryologically lethal [31], mice deficient in both TNF-α receptors (*TNF p55^−/−^ p75^−/−^*) and their WT littermates were used and subjected to either a single dose of CCl_4_ or mineral oil, and sacrificed after 24 h to evaluate hepatic injury. Liver sections from *TNF p55^−/−^ p75^−/−^* mice revealed a marked reduction of necroinflammatory injury, compared to WT controls (**Figure 7E**). These findings were corroborated by a marked, 80% reduction of serum ALT levels in *TNF p55^−/−^ p75^−/−^* mice, compared to CCl_4_-injected WT controls (4,581 IU/L and 22,660 IU/L, respectively, *P*≤0.05; **Figure 7F**). This implicates the involvement of TNF-signaling in CCl_4_-induced hepatic injury and provides a mechanistic explanation for the hepatoprotective effects of Marimastat in both acute, and chronic CCl_4_-induced hepatic injury.

## Discussion

**Figure 8:**
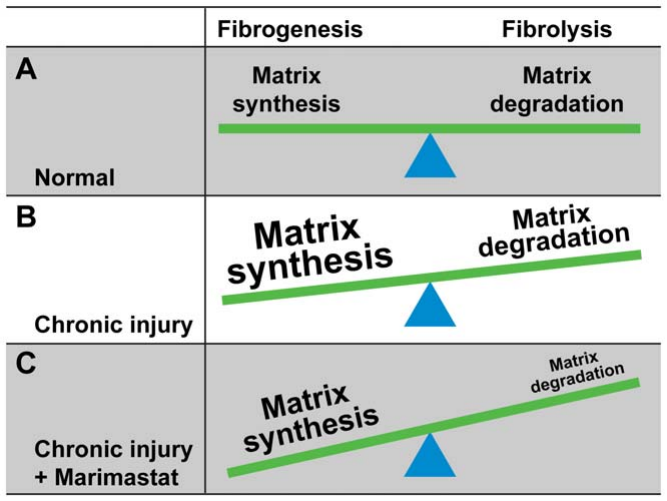
Broad-spectrum matrix metalloproteinase (MMP)-inhibition results in increased hepatic fibrosis following chronic hepatic injury. In the absence of liver injury a physiological balance exists between extracellular matrix synthesis and its degradation (A). Chronic hepatic injury causes excessive synthesis of extracellular matrix proteins including collagen, which prevails over their enzymatic degradation resulting in liver fibrosis (B). Despite a significant attenuation of fibrogenesis and inflammation, efficient inhibition of fibrolytic matrix metalloproteinases by a broad spectrum MMP-inhibitor has profound effects on collagen degradation, tilting the balance towards net extracellular matrix deposition and scar tissue formation (C).

The present study was aimed to determine the effects of the broad spectrum MMP-inhibitor Marimastat on fibrosis formation in a murine model of repeated, chronic CCl_4_-induced hepatic injury. We demonstrate that pharmacologic pan-MMP inhibition very efficiently decreased hepatic injury by amelioration of the inflammatory response and by downregulation of pro-fibrogenic mRNA expression, through interfering, at least in part, with TNF-α activation and signaling. The detrimental effects of MMP-inhibition on scar formation, however, were unexpected. We initially hypothesized that inhibition of MMP activity would lead to a further decrease in hepatic injury, rather than impacting on collagen accumulation, resulting in decreased fibrosis formation. Obviously, broad spectrum MMP-inhibition, as determined by MMP transcript levels and activity assays, counterbalanced the potential beneficial anti-fibrogenic and anti-inflammatory effects by efficient inhibition of fibrolysis (**Figure 8**). This dissociation between inflammation and liver injury on the one hand, and fibrolysis on the other hand has to our knowledge not been described previously and may provide novel insights in the dual role of MMPs in fibrogenesis, and fibrolysis.

MMPs are secreted as zymogens and become activated by cleavage of their propeptide [32], [33]. Marimastat is a synthetic, low molecular weight succinyl hydroxamate that inhibits MMPs via its hydroxamate group that complexes the zinc ion needed in the active site of MMPs [27]. Marimastat also inhibits TACE, with a suggested benefit in diseases that involve both inflammation and extracellular matrix remodeling [34]. Although MMPs were traditionally viewed as molecules that were only involved in degradation and turnover of the extracellular matrix, novel insights overturned this dogma and revealed that MMPs modulate the activities of a wide range of extracellular and intracellular signaling pathways [22], [23]. By regulating cell proliferation, migration, adhesion, growth factor bioavailability, chemotaxis, and cell signaling, MMPs are crucial for physiological and pathophysiological processes such as inflammation, immunity, angiogenesis, tumorigenesis, metastasis, and wound healing. As a consequence, a broad spectrum MMP inhibitor such as Marimastat was expected to have both anti-inflammatory and potential antifibrotic properties.

It has been generally accepted that changes in patterns of matrix degradation are critical for fibrogenesis [35]; however, the role of MMP activity in the liver during fibrogenesis is not yet fully understood. In rodents, MMP-2, MMP-3, MMP-9, MMP-13, MMP-14, as well as TIMP-1 and TIMP-2 are expressed in early stages of HSC activation and have been implicated in fibrogenesis as well as fibrolysis (reviewed in [20]). We confirmed that the broad-spectrum MMP-inhibitor Marimastat efficiently inhibited gelatinolytic and collagenolytic MMP, as well as TACE activities. Among other pro-fibrogenic genes, hepatic transcript levels of α-SMA were significantly decreased in the Marimastat treated animals suggesting decreased activation of a subset of HSCs and ameliorated fibrogenesis. This was confirmed by immunohistochemical staining for α-SMA followed by quantification of positive cell counts. It must be noted, however, that there exists heterogeneity of gene expression in myofibroblastic cells during fibrogenesis which reduces the usefulness of α-SMA as a marker for collagen production [36]. MMPs such as MMP-8, MMP-9, and MMP-13 possess the ability to degrade the extracellular matrix by breakdown of fibrillar collagen type I [9], [19], [37]. MMP-9 may indirectly contribute to fibrolysis by accelerating HSC apoptosis, whereas MMP-2 may rather drive hepatic stellate activation and unfavorable basement membrane remodeling resulting in more fibrosis [20], [38]. We demonstrated efficient inhibition of gelatinolytic and collagenolytic MMP-activities which as a whole has shifted the balance towards a net accumulation of hepatic fibrosis.

TIMP-1 is the most relevant physiological MMP-inhibitor in fibrotic diseases, including hepatotoxic and cholestatic injury, whose expression is upregulated by various inflammatory cytokines [32], [33]. Antagonizing TIMP-1 using both neutralizing antibodies and gene therapy, as well as indirect TIMP-1 mRNA reduction by antagonizing inflammatory cytokines improved experimental fibrosis in rodents [20]. In addition, transgenic mice overexpressing human TIMP-1 developed more liver fibrosis when subjected to chronic CCl_4_ administration [39], and demonstrated attenuated spontaneous fibrosis resolution [40]. Reduced TIMP-1 levels may also accelerate hepatocyte proliferation following partial hepatectomy, illustrating its inhibitory role in hepatocyte regeneration [41]. In our study, inflammation was significantly decreased by TACE inhibition while downregulation of TIMP-1 may have further improved hepatic regeneration, as reflected by the marked decrease in serum levels of alkaline phosphatase and ALT.

To further elucidate the hepatoprotective effects of broad spectrum MMP-inhibition, we dosed Marimastat-pretreated C57Bl/6J mice with a single injection of CCl_4_, to demonstrate reduced centrilobular necrosis and a marked (57%) reduction in serum ALT levels corroborating the hepatoprotective effects of Marimastat also in acute liver failure. These data are in line with a previous report describing the use of a similar broad-spectrum MMP-inhibitor (Batimastat; BB-94, British Biotech) in the prevention of acute, fulminant hepatitis induced by TNF-α combined with d-(+)-galactosamine [28]. In this study, mice treated with BB-94 as well as mice deficient in *Mmp2*, *Mmp3* or *Mmp9* had lower levels of apoptosis and necrosis of hepatocytes, and better survival. Although TACE inhibitors are efficient in protecting against lipopolysaccharide/d-(+)-galactosamine-induced lethal hepatitis by inhibition of TNF-α release, inhibition of TACE by BB-94 was deemed irrelevant in their model; however, data was not shown [28]. The authors speculated that the absence or presence of endogenous TNF-α does not influence the outcome after TNF-α/d-(+)-galactosamine challenge [28].

Observations that soluble TNF-α receptor treatment improved the outcome following acute CCl_4_-intoxication [42], and that monoclonal antibodies against TNF-α improved experimental CCl_4_-induced fibrosis [43] led us to explore the involvement of TNF-α and TACE in CCl_4_-mediated hepatotoxicity. We show that after a single dose of CCl_4_, animals pretreated with the broad-spectrum MMP- and TACE-inhibitor Marimastat, as well as animals deficient in both TNF-α receptors (p55 and p75) were markedly protected, as demonstrated by attenuated necroinflammatory injury on histology and 5-fold lower serum ALT levels, compared to their wild type controls. These findings indicate the pivotal role of TNF-α signaling in CCl_4_ mediated hepatotoxicity. A previous study failed to demonstrate a significantly ameliorated response of *TNF p55^−/−^ p75^−/−^* mice to acute CCl_4_ intoxication [44], which is in contrast with our results. But these authors and others found reduced fibrogenesis following repeated CCl_4_-administration in animals lacking the TNF-α p55 receptor supporting the involvement of TNF-signaling in CCl_4_-mediated hepatotoxicity [44], [45]. These data suggest that specific inhibition of TACE may be an attractive approach to manipulate the inflammatory cascade following a hepatic insult.

We also explored the possible involvement of MMP-9 in the protection against acute CCl_4_-induced injury. A previous report showed that acute CCl_4_-induced liver injury in rats increased both active and latent MMP-9 to maximum levels at 24 h and remained elevated 3 days following the injection, suggesting its involvement in early hepatic injury [46]. We show that after a single dose of CCl_4_ animals deficient in *Mmp9* exhibited similar hepatic injury compared to their wild type controls, as assessed by histology and serum ALT levels after 24 h. The interpretation of results with MMP knockout mice, however, is difficult since the net proteolytic activity of MMPs relies upon complex, direct interactions between the different protease and protease inhibitor families. In addition, adaptive upregulation of gelatinolytic/collagenolytic activities can have occurred, which would necessitate a conditional knockout. Nevertheless, the almost identical results of both *Mmp9^−/−^* animals and their wild type controls largely rules out a major role of MMP-9 in the protection against acute CCl_4_ intoxication.

More recently, another paper described attenuation of liver injury following treatment with the MMP-inhibitor CTS-1027 (Conatus Pharmaceuticals, San Diego, CA, USA)[47]. Using the bile duct ligation model, a decrease in hepatocyte apoptosis and a reduction in markers for HSC activation and fibrogenesis was demonstrated, which is in line with our results that Marimastat attenuated hepatic inflammation and necrosis coupled with downregulation of genes related to fibrogenesis. CTS-1027 is actually now being further evaluated as a potential drug in patients with Hepatitis C Virus [48]; however, we believe that in light of our results caution is warranted. Although it was demonstrated that two weeks of treatment with CTS-1027 resulted in attenuation of collagen deposition in the bile duct ligation model, we found that inhibition of MMP- and TACE-activity with Marimastat during chronic CCl_4_ administration for 6 weeks resulted in increased net fibrosis. It is well possible that two weeks of MMP-inhibition may attenuate injury without aggravating fibrosis, while longer-term treatment in the presence of ongoing liver injury may shut down matrix degradation, eventually leading to impaired fibrolysis. Although we cannot extrapolate our results with Marimastat to all MMP inhibitors and potential off-target effects of Marimastat may have contributed to our findings, it is likely that long-term use of all such inhibitors with anti-TACE activity will have similar divergent effects on liver injury and fibrosis.

Clinically, Marimastat has been used in multiple oncologic clinical trials up to phase III; however, its overall therapeutic benefit was limited and the trials were halted [49]–[51]. The most consistently reported side effect was musculoskeletal pain, likely due to inhibition of adaptive ECM remodeling; however, spontaneous fibrosis formation has never been reported. This suggests that in the absence of chronic hepatic injury such as hepatitis, or non-alcoholic steatohepatitis, broad-spectrum MMP-inhibition is unlikely to be harmful to the liver. This may also apply to other MMP-inhibitors such as the commonly prescribed antibiotic doxycycline [52], [53]. Nonetheless, we believe that caution is warranted when patients with chronic, active liver diseases receive MMP-inhibitors, since inhibition of fibrolytic MMPs will likely accelerate hepatic fibrogenesis formation.

In conclusion, we demonstrate that broad-spectrum MMP- and TACE-activity inhibition with Marimastat during chronic CCl_4_ administration resulted in significantly attenuated hepatic inflammation and necrosis coupled with downregulation of genes related to fibrogenesis, but resulted in increased liver fibrosis. Inhibition of MMPs and collagen degradation by Marimastat, however, counterbalanced the beneficial anti-inflammatory effect, resulting in a positive balance of collagen deposition. Since effective inhibition of fibrolytic activity by MMPs accelerates fibrosis progression, our data suggests a note of caution for the use of broad-spectrum MMP inhibitors in patients with chronic, ongoing liver diseases, or for the treatment of liver fibrosis itself. Specific inhibition of TACE, however, may still be an attractive approach to manipulate the inflammatory cascade following a hepatic insult.

## Methods

### Ethics statement

Animal protocols complied with the National Institutes of Health Animal Research Advisory Committee guidelines and were approved by the Children's Hospital Boston Animal Care and Use Committee (protocol no. A06-08-065R).

### Animals

Male 6-week-old C57BL6/J mice (Jackson Laboratories, Bar Harbor, ME) were housed five per cage on paper chip bedding in a barrier room with regulated temperature (21°C±1.6°C), humidity (45%±10%), and an alternating 12-hour light and dark cycle. The animals had free access to water and standard American Institute of Nutrition (AIN) 93 M (TD.94048; Harlan Teklad, Madison, WI) purified rodent diet throughout the study.

### Animal experiments

**Figure 9:**
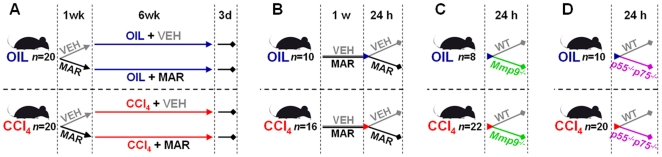
Design of the studies. Two groups of C57Bl/6J mice were randomized to two subgroups to receive either Marimastat or methylcellulose vehicle twice daily (A). Liver fibrosis was induced by repeated carbon tetrachloride (CCl4) administration for 6 weeks in one group of animals, whereas a second group of control animals received the mineral oil vehicle alone (A). The protective effects of Marimastat were further evaluated in a model of acute CCl4-induced hepatotoxicity (B). C57Bl/6J mice received either Marimastat or methylcellulose vehicle twice daily for 1 week, after which they were subjected to a single dose of CCl4 or mineral oil as control (B). The mechanism was further elucidated by subjecting Mmp9−/− mice and their wild type (WT) littermates (C), or TNF p55−/−p75−/− mice and their WT littermates (D) to either a single dose of CCl4, or mineral oil. In all acute CCl4-experiments, animals were sacrificed after 24 hours. Oil, non-fibrotic control group; CCl4, fibrotic mice; VEH, vehicle treated control group; MAR, Marimastat treated experimental group; WT, wild type.

After one week of acclimation, 40 C57BL6/J mice were randomized into 4 groups (10 mice each). The first week, two groups received 100 mg/kg of Marimastat (BB-2516, British Biotech, UK) in 0.45% methylcellulose (Sigma-Aldrich, St. Louis, MO) vehicle twice daily via orogastric gavage (MAR), and two groups received vehicle alone (VEH). These treatments were continued until study completion. Marimastat efficiently inhibits MMP-2, MMP-3, MMP-7, MMP-9, MMP-13 and TACE activity, with IC_50_s in the nM range [26], [54]. After one week of treatment with either Marimastat or vehicle, animals received three intragastric doses via oral gavage (Monday, Wednesday and Friday) of CCl_4_ (anhydrous, ≥99.5%, Sigma-Aldrich, St. Louis, MO) dissolved in mineral oil (Sigma-Aldrich, St. Louis, MO) or vehicle alone each week for another six weeks. The initial dose was 0.875 mL/kg, followed by 8 doses of 1.75 mL/kg and 9 doses of 2.5 mL/kg, respectively. At the end of the seven week treatment period and three days after the last dose of CCl_4_, animals were sacrificed to evaluate hepatic fibrosis and related parameters (**Figure 9A**).

In separate experiments, the effect of MMP inhibition on acute liver injury was studied in 64 C57BL6/J mice after orogastric administration of a single dose of 1.5 mL/kg CCl_4_. Starting one week prior to the hepatotoxic challenge, 32 mice received 100 mg/kg of Marimastat twice daily via orogastric gavage (MAR), and 32 mice received vehicle alone (VEH). Treatment was continued until sacrifice. Animals were sacrificed 12 h, 24 h, 48 h and 96 h after CCl_4_ administration (*n* = 8 per treatment and time point) to evaluate hepatic injury (**Figure 9B**). Since the peak of hepatic injury occurred after 24 h, this time point was chosen as surrogate endpoint in subsequent experiments.

The contribution of MMP and TACE-dependent pathways was studied using mice deficient in MMP-9, as well as mice deficient in both TNF-α receptors. Thirty mice homozygous null for the *Mmp9* gene (B6.FVB(Cg)-*Mmp9^tm1Tvu^*/J mice; Jackson Laboratories, Bar Harbor, ME) and their wild type (WT) littermates, and 30 mice homozygous for both TNF-α receptor I (*Tnfrsf1a^tm1Imx^*; p55) and TNF-α receptor II (*Tnfrsf1b^tm1Imx^*; p75) null mutations (B6;129S-*Tnfrsf1a^tm1Imx^ Tnfrsf1b^tm1Imx^*/J; Jackson Laboratories, Bar Harbor, ME) and their WT littermates were subjected to either a single dose of 1.5 mL/kg CCl_4_ via orogastric gavage, or mineral oil (**Figures 9C, D**). Animals were sacrificed after 24 h to evaluate hepatic injury.

### Sample collection and serum biochemistry

Animals in all groups were euthanized, and pieces of their livers fixed in 10% formalin for histology or immediately frozen and stored for RNA extraction and hydroxyproline measurements. Blood was collected via retro-orbital sinus puncture and plasma was obtained via centrifugation. Serum was frozen at -80°C for analysis of alanine aminotransferase (ALT) and alkaline phosphatase (AP) activities at the Clinical Laboratory of Children's Hospital Boston. Serum levels of IL-6 and the soluble TNF-α receptor II (p75) were determined using a commercial ELISA kit (Quantikine, R&D Systems, Minneapolis, MN). Optical density was read at 450 nm and analyzed with Softmax PRO Software (Molecular Devices, Sunnyvale, CA).

### Histology

Paraffin-embedded sections from the frontal lobes of the liver were stained by hematoxylin and eosin (H&E) to examine cellular architecture and lipid accumulation. A pathologist (VN) blinded to the treatment groups conducted a histological analysis of the liver sections [55]. Lobular inflammation was quantified by assessing the number of inflammatory foci per microscopic field. Five fields were checked at 200× magnification as follows: 0 (absent), 1 (<2 foci), 2 (2–4 foci), and 3 (>4 foci). Steatosis was scored by the percentage (%) of liver cells containing fat: 0 (<5%), 1 (5–33%), 2 (>33–66%) and 3 (>66%). Necrosis was scored as 0 (absent) or 1 (pericentral area occupied by necrosis).Masson trichrome (MT) and Sirius Red stains of paraffin-embedded sections were used to qualitatively assess collagen architecture and extent of fibrosis. Morphometric analysis for fibrosis quantification was performed using ten random high power fields (HPF) per animal at 200× magnification. These images were quantified using NIH ImageJ software (http://rsb.info.nih.gov/ij/).

### Immunohistochemistry

Immunohistochemistry was performed using 4 µm thick formalin-fixed, paraffin-embedded tissue sections. Briefly, slides were soaked in xylene, passed through graded alcohols and put in distilled water. Slides were then pre-treated with 1.0-mM EDTA, pH 8.0 (Zymed, South San Francisco, CA) for anti-CD3 or Citrate buffer for anti-alpha smooth muscle actin (α-SMA) in a steam pressure cooker (Decloaking Chamber, BioCare Medical, Walnut Creek, CA) as per manufacturer's instructions followed by washing in distilled water. All further steps were performed at room temperature in a hydrated chamber. Slides were pre-treated with Peroxidase Block (DAKO USA, Carpinteria, CA) for 5 minutes to quench endogenous peroxidase activity. For CD3, polyclonal rabbit anti-murine CD3 antibody (Cell Marque, Rocklin, CA. Cat #CMC363) was applied 1∶1500, and for α-SMA, rabbit anti-murine α-SMA (Abcam, Cambridge, MA, Cat #ab5694) was applied 1∶200 in diluent (DAKO) for 1 hour. Slides were washed in 50-mM Tris-Cl, pH 7.4, and antigens detected with anti-rabbit Envision+ kit (DAKO) as per manufacturer's instructions. After further washing, immunoperoxidase staining was developed using a DAB chromogen (DAKO) and counterstained with hematoxylin. For F4/80 staining, slides were incubated with proteinase K applied 1∶5 in DAKO diluent for 10 minutes and then washed in 50-mM Tris-Cl, pH 7.4, followed by incubation with rat F4/80 antibody (Serotec, Raleigh, NC, cat# MCA497GA) applied 1∶10,000 in DAKO diluent for 1 hour. Slides were then washed and incubated with rabbit anti-rat secondary (DAKO) diluted 1∶750 for 30 minutes, washed, and detected with anti-rabbit Envision+ kit (DAKO) as described above. For quantification purposes, positive cells were counted in ten random HPF per animal at 200× magnification and expressed as mean positive cells/10 HPF.

### Liver hydroxyproline determination

Hepatic collagen content was quantified biochemically by determining liver hydroxyproline using an established method with minor modifications [56]–[58]. Briefly, snap-frozen liver tissue from two different lobes (50–60 mg each) was hydrolyzed at 110°C for 16 h in 5 mL 6N HCl. The hydrolysate was filtered, 50-µL aliquots were evaporated under vacuum, and the sediment was dissolved in 1.2 mL of 50% isopropanol and incubated with 0.2 mL of 0.84% chloramine-T in 42 mmol/L sodium acetate, 2.6 mmol/L citric acid, and 39.5% (vol/vol) isopropanol (pH 6.0), followed by incubation for 10 minutes at room temperature. Next, 0.248 g *p*-dimethylaminobenzaldehyde, dissolved in 0.27 mL of 60% perchloric acid, and 0.73 mL isopropanol were added and incubated at 50°C for 90 minutes. Relative hydroxyproline (µg/g liver) was then quantified photometrically at 558 nm and total hydroxyproline (mg/whole liver) was calculated based on individual liver weights and the corresponding relative hydroxyproline content from representative liver samples, as established previously [57], [58].

### Quantitative Real-Time RT-PCR

200–300 mg snap-frozen liver tissue from two lobes was homogenized; total RNA was extracted using Tri Reagent (Molecular Research Center, Cincinnati, OH) and reverse transcribed as described [58], [59]. Relative transcript levels were quantified by real-time RT-PCR on a LightCycler 1.5 instrument (Roche, Mannheim, Germany) using the TaqMan methodology as described in detail [57]–[59]. TaqMan probes (dual-labeled with 5′-FAM and 3′-TAMRA) and primers were designed based on published sequences (**Table 1**) using the Primer Express software (Perkin Elmer, Wellesley, USA), synthesized at MWG Biotech AG (Ebersberg, Germany), and are published elsewhere [57], [59]. The housekeeping gene beta-2 microglobulin (β2MG) was amplified in parallel reactions for normalization.

**Table 1:** Primers and probes used in quantitative real-time RT-PCR.

Target gene	5′-Primer	TaqMan probe	3′-Primer
**Procollagen α1(I)** [NM_007742]	TCCGGCTCCTGCTCCTCTTA	TTCTTGGCCATGCGTCAGGAGGG	GTATGCAGCTGACTTCAGGGATGT
**β6 Integrin** [NM_021359]	GCAGAACGCTCTAAGGCCAA	TGGCAAACGGGAACCAATCCTCTGT	AAAGTGCTGGTGGAACCTCG
**TGF-β1** [NM_011577]	AGAGGTCACCCGCGTGCTAA	ACCGCAACAACGCCATCTATGAGAAAACCA	TCCCGAATGTCTGACGTATTGA
**TGF-β2** [NM_009367]	GTCCAGCCGGCGGAA	CGCTTTGGATGCTGCCTACTGCTTTAGAAAT	GCGAAGGCAGCAATTATCCT
**α-SMA** [NM_007392]	ACAGCCCTCGCACCCA	CAAGATCATTGCCCCTCCAGAACGC	GCCACCGATCCAGACAGAGT
**TIMP-1** [NM_001044384]	TCCTCTTGTTGCTATCACTGATAGCTT	TTCTGCAACTCGGACCTGGTCATAAGG	CGCTGGTATAAGGTGGTCTCGTT
**TNF-α** [NM_013693]	GGGCCACCACGCTCTTC	ATGAGAAGTTCCCAAATGGCCTCCCTC	GGTCTGGGCCATAGAACTGATG
**MMP-2** [NM_008610]	CCGAGGACTATGACCGGGATAA	TCTGCCCCGAGACCGCTATGTCCA	CTTGTTGCCCAGGAAAGTGAAG
**MMP-3** [NM_010809]	GATGAACGATGGACAGAGGATG	TGGTACCAACCTATTCCTGGTTGCTGC	AGGGAGTGGCCAAGTTCATG
**MMP-8** [NM_008611]	CAGGGAGAAGCAGACATCAACA	TGCTTTCGTCTCAAGAGACCATGGTGAC	GATTCCATTGGGTCCATCAAA
**MMP-9** [NM_013599]	CAGGATAAACTGTATGGCTTCTGC	CTACCCGAGTGGACGCGACCGT	GCCGAGTTGCCCCCA
**MMP-13** [NM_008607]	GGAAGACCCTCTTCTTCTCT	TCTGGTTAACATCATCATAACTCCACACGT	TCATAGACAGCATCTACTTTGTT
**β2MG**	CTGATACATACGCCTGCAGAGTTAA	GACCGTCTACTGGGATCGAGACATGTG	ATGAATCTTCAGAGCATCATGAT

### Gelatinase and Interstitial Collagenase Activity Assays

Determination of gelatinase and interstitial collagenase activity was performed as described previously with assays based on degradation of DQ-gelatin and DQ-collagen type I, respectively (Molecular Probes Inc., Eugene, OR) [58], [59]. DQ-substrates are heavily labeled with FITC which quenches their fluorescence, but fluorescent peptides are generated upon proteolytic cleavage. 20 µL of 10% liver homogenates prepared as described previously[57] were diluted in a total volume of 200 µL of MMP-activity buffer (50 mM Tris-HCl, 150 mM NaCl, 5 mM CaCl2, 0.025% Brij 35™, pH 7.5, supplemented with EDTA-free protease inhibitor cocktail (Complete™, Roche Applied Science, Mannheim, Germany)) and incubated with DQ-substrates (25 µg/mL) and increasing doses of Marimastat dissolved in H_2_O at room temperature for 1–20 h in 96-well plates in triplicates. Fluorescence was measured using the Wallac Victor2 Multilabel Counter (PerkinElmer, Inc., Waltham, MA); with excitation at 485 nm, and emission at 535 nm. Human recombinant activated MMP-1 (collagenase) and MMP-2 (gelatinase) were used as positive controls. Since all procedures including extraction were performed in the presence of protease inhibitors to prevent *ex vivo* MMP activation, data obtained represent the net endogenous gelatinolytic and collagenolytic activities.

### Statistical analysis

Data are expressed as means ± standard error of the mean (SEM). Data sets involving more than two groups were assessed by analysis of variance (ANOVA). Differences between two groups were assessed using the unpaired two-tailed Student's *t* test, or if nonparametric, by using the Mann-Whitney *U* test. *P*≤0.05 was considered statistically significant. All data were collected in a computerized Microsoft Excel database (Microsoft Inc., Redmond, WA). The analysis was performed with SPSS version 16.0 (SPSS Inc., Chicago, IL) statistical software, and figures were created using GraphPad Prism version 5.0 (GraphPad Software Inc., La Jolla, CA) software.
